# Non-Cavitation Targeted Microbubble-Mediated Single-Cell Sonoporation

**DOI:** 10.3390/mi13010113

**Published:** 2022-01-11

**Authors:** Xiufang Liu, Wenjun Zhang, Yanshu Jing, Shasha Yi, Umar Farooq, Jingyao Shi, Na Pang, Ning Rong, Lisheng Xu

**Affiliations:** 1 College of Medicine and Biological Information Engineering, Northeastern University, 195 Innovation Road, Shenyang 110016, China; xf.liu@siat.ac.cn (X.L.); na.pang@siat.ac.cn (N.P.); 2 Paul C. Lauterbur Research Center for Biomedical Imaging, Shenzhen Institutes of Advanced Technology, Chinese Academy of Sciences, Shenzhen 518055, China; jing_ys@stu.xjtu.edu.cn (Y.J.); ss.yi@siat.ac.cn (S.Y.); farooq@siat.ac.cn (U.F.); jy.shi@siat.ac.cn (J.S.); 3 Department of Mechanical and Electrical Engineering, Gannan University of Science and Technology, 156 Kejia Avenue, Ganzhou 341000, China; askzwj@gmail.com; 4 Department of Biomedical Engineering, School of Life Science and Technology, Xi’an Jiaotong University, Xi’an 710049, China; 5 Neusoft Research of Intelligent Healthcare Technology, Co., Ltd., Shenyang 110167, China

**Keywords:** sonoporation, TMBs, ARF, input voltage

## Abstract

Sonoporation employs ultrasound accompanied by microbubble (MB) cavitation to induce the reversible disruption of cell membranes and has been exploited as a promising intracellular macromolecular delivery strategy. Due to the damage to cells resulting from strong cavitation, it is difficult to balance efficient delivery and high survival rates. In this paper, a traveling surface acoustic wave (TSAW) device, consisting of a TSAW chip and a polydimethylsiloxane (PDMS) channel, was designed to explore single-cell sonoporation using targeted microbubbles (TMBs) in a non-cavitation regime. A TSAW was applied to precisely manipulate the movement of the TMBs attached to MDA-MB-231 cells, leading to sonoporation at a single-cell level. The impact of input voltage and the number of TMBs on cell sonoporation was investigated. In addition, the physical mechanisms of bubble cavitation or the acoustic radiation force (ARF) for cell sonoporation were analyzed. The TMBs excited by an ARF directly propelled cell membrane deformation, leading to reversible perforation in the cell membrane. When two TMBs adhered to the cell surface and the input voltage was 350 mVpp, the cell sonoporation efficiency went up to 83%.

## 1. Introduction

The delivery of membrane-impermeant compounds into living cells for molecular biology and gene therapy is a critical step in clinical and research applications, which can be achieved by using carrier-based and membrane disruption-based techniques [1]. Carrier-based methods mainly depend on endocytosis and fusogenic activity [2,3], endowing the carrier with the ability to merge directly with the cell membrane. Due to the relatively low efficiency of carrier-based methods at delivering membrane-impermeant compounds, membrane disruption methods have attracted increasing attention. Recently, physical approaches, such as mechanical, optical, thermal, electrical, magnetic, and acoustic approaches, have been widely exploited for membrane disruption [1,4,5,6,7,8,9]. Because there is high biocompatibility, no contact, and relative safety, and because it is label free, the acoustic technique for mediating membrane disruption (also called sonoporation) is popularly applied in the delivery of membrane-impermeant compounds.

Sonoporation employs ultrasound to generate transient pores in the cell membrane, and is a promising means of delivering membrane-impermeant compounds [10,11,12,13,14]. Several studies have demonstrated that sonoporation efficiency can be improved by introducing microbubbles (MBs) and that sonoporation is not likely to occur in the absence of MBs [15]. MBs are composed of an inert gas (such as SF_6_, C_3_F_8_, or C_4_F_10_) and a shell made of lipids, polymers, or proteins [16,17]. Targeted MBs (TMBs) that adhere to cells or tissues through specific binding means are promising agents for drug delivery. MBs usually serve as cavitation nuclei to achieve sonoporation. During sonoporation, pores can be produced by the mechanical forces resulting from MB cavitation [12,13,18]. When exposed to low acoustic pressure, MBs undergo repeated symmetric linear oscillation for stable (non-inertial) cavitation. In a high-intensity acoustic field, MBs oscillate asymmetrically and then collapse and fragmentate violently, a process known as inertial cavitation. The collapse leads to jetting, shock waves, and temperature elevation, severely damaging cells. Compared to inertial cavitation, stable cavitation is moderate and controllable for cell sonoporation, while the efficiency is relatively low. 

Due to the absorption, scattering, and reflection of acoustic waves by MBs, momentum and energy can be exchanged between the MBs and acoustic waves, causing an acoustic radiation force (ARF), which is responsible for the transfer of MBs [19,20]. Several studies have indicated that the ARF can induce MBs to move in the direction of the ultrasound beam. Dayton et al. verified that the translational movement of MBs is mainly attributed to the ARF [21]. Zhou et al. also showed that MBs use the ARF to move toward cells [22]. Thus, the ARF acts on the TMBs bonded to adherent cells, making them move, and may directly cause cell deformation via pulling and pushing forces, leading to cell sonoporation. 

With the development of microelectro-mechanical system (MEMS) techniques, acoustofluidic devices are being increasingly used to regulate cell membrane permeability and explore ultrasound bioeffects. Carugo et al. indicated that the ARF induces cell sonoporation in the absence of MBs and retains high cell viability [23]. Meng et al. found that an ARF can direct MBs toward the cell surface and eventually realize cell sonoporation at the single-cell level [11]. Single-cell sonoporation is crucial for understanding the physical mechanisms of pore formation. Therefore, an acoustofluidic device may help researchers investigate the mechanism of non-cavitation, TMB-mediated single-cell sonoporation.

**Figure 1:**
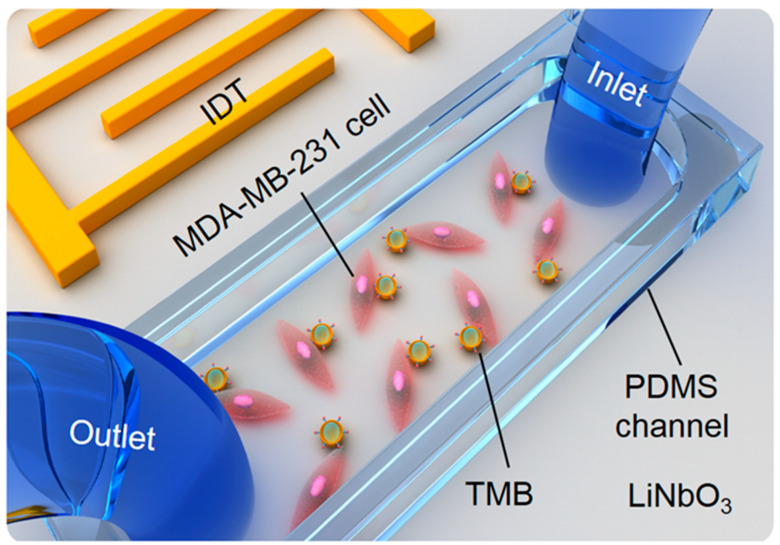
Schematic of the experimental device. The MDA-MB-231 cells were cultured in a PDMS channel, and the TMBs adhered to cells. The TSAW was applied to excite TMBs for cell sonoporation.

In this paper, we designed a TSAW device to study the single-cell sonoporation induced by non-cavitation TMBs adhering to the MDA-MB-231 cell surface, as shown in Figure 1. When the TSAW excited the TMBs, the cell was deformed by the ARF acting on the TMBs, and pores were formed in the cell membrane. Laser Doppler vibration (LDV) based on a passive cavitation detector (PCD) method verified that bubble cavitation did not occur. Moreover, when the input voltage was 350 mVpp and two TMBs were attached to the cells, the sonoporation efficiency went up to 83%. This TSAW device may provide a new strategy for intracellular delivery and gene therapy in a non-viral and label-free manner.

## 2. Materials and Methods

### 2.1. The Fabrication of the TSAW Device

The TSAW device was composed of a TSAW chip and a polydimethylsiloxane (PDMS, Sylgard 184, Dow Corning, Wiesbaden, Germany) channel. This TSAW chip was designed and fabricated according to a standard microfabrication technique [24,25,26,27]. A periodic array of interdigital transducers (IDTs) was deposited on the surface of a 128° Y–*X*-axis-rotated cut, X-propagating lithium niobate (LiNbO_3_) crystal substrate with a gold layer of 200 nm. The propagation velocity of the SAW was 3900 m/s, and the center frequency was 24 MHz. Correspondingly, the wavelength of the SAW was 160 μm.

A PDMS channel 40 μm in height was designed using standard soft lithograph and mold replica techniques [14,28,29]. Briefly, a layer of negative SU-8 photoresist was spin-coated on the surface of a silicon wafer substrate. After being exposed to UV light and baking, a pattern mask was transferred as a solid mold onto the silicon wafer substrate. The mixture of the PDMS prepolymer and curing agent at a 1:10 curing ratio was poured onto a patterned silicon wafer and baked at 80 °C for 60 min. Then, the PDMS channel was peeled off from the silicon wafer. The inlet and outlet of the PDMS channel were fabricated using a 0.75 mm puncher (Harris Uni-Core, World Precision Instruments, Sarasota, FA, USA). Subsequently, the PDMS channel was boned to the TSAW chip permanently following oxygen plasma treatment (150 W, 2 min), as shown in Figure 1.

### 2.2. Cell Culture in the PDMS Channel

The human triple-negative breast cancer cell line, MDA-MB-231, was purchased from the cell bank of the Chinese Academy of Science. Dulbecco’s Modified Eagle Medium (Gibco, Life Technologies, Carlsbad, CA, USA) containing 10% fetal bovine serum (FBS, Gibco, Auckland, NE, USA), 1% penicillin-streptomycin, and 1% glutamine was applied for culturing the MDA-MB-231 cells. The cells were cultured in an incubator containing 5% CO_2_ at 37 °C. When the cell confluence reached 96%, the cells were collected via 0.25% trypsin digestion and the cell density was adjusted to 1 × 10^7^ cells/mL.

Before the cells were seeded in the PDMS channel, Poly-L-lysine (Sigma-Aldrich, St. Louis, MO, USA) was injected into the PDMS channel using a syringe with a PTFE tube. Then, the PDMS channel was incubated in an incubator at 37 °C for 30 min. Subsequently, the PDMS channel was washed 3 times with a phosphate buffer and exhausted with a vacuum pump for 30 min. 

The MDA-MB-231 cell suspension with a density of 1 × 10^7^ cells/mL was injected into the PDMS channel using a sterile syringe. The whole process of cell injection was observed under a microscope. The number of cells was controlled to grow suitably in the PDMS channel. After the injection, the TSAW device was placed in an incubator with 5% CO_2_ at 37 °C for 12–16 h to ensure that the cells fully adhered to the surface of LiNbO_3_. 

### 2.3. Adhesion of Cells and TMBs

Biotinylated microbubbles were prepared as previously described [30,31]. Briefly, distearoyl phosphatidylcholine (1,2-distearoyl-sn-glycero-3-phosphocholine, DSPC, Avanti, Alabaster, AL, USA), distearoyl phosphatidylethanolamine-polyethylene glycol 2000 (1,2-distearoyl -sn-glycero-3-phosphoethanolamine-N-[methoxy(polyethylene glycol)-2000], DSPE-PEG 2000, Avanti, Alabaster, AL, USA), and distearoylphosphatidylethanolamine-polyethylene glycol 2000-biotin (1, 2-distearoyl-sn-glycero-3-phosphoethanolamine-N-[biotinyl(polyethylene glycol)-2000)] (ammonium salt), DSPE-PEG2000-Biotin, Avanti, Alabaster, AL, USA) were dissolved in chloroform, and the final concentrations were 20 mg/mL, 18 mg/mL, and 10 mg/mL, respectively. Then, the three solutions were mixed at a molar ratio of 18:1:1 to evaporate under nitrogen flow to generate a thin layer of phospholipid membrane. After 4 h of vacuum treatment, the phospholipid membrane was hydrated with Tris (hydroxymethyl) aminomethane buffer saline at 65 °C and then transferred into vials of 1 mL solution. The perfluoropropane (Flura, Newport, TN, USA) was injected into the vials without any air. The prepared biotinylated microbubbles were stored at 4 °C. 

By applying the specific binding characteristics of biotin–avidin–biotin, the TMBs specifically targeting MDA-MB-231 cells were prepared. The biotinylated microbubbles were mechanically vibrated for 30 s and then incubated with avidin for 30 min at room temperature. After removing the free avidin via centrifuging, the biotinylated microbubbles of 5 × 10^8^ were further incubated with 30 μg biotinylated Piezo-1 antibody (NOVUS, Littleton, CO, USA) to obtain the TMBs for MDA-MB-231. The TMBs were stored in a refrigerator at 4 °C and sealed for subsequent experiments.

After the cells in the PDMS cavity were fully adherent and grown, an appropriate amount of TMBs was injected into the PDMS cavity and incubated for 15 min at room temperature. The TMBs adhered to the cell surface through the specific binding of antibodies and antigens.

### 2.4. Cell Sonoporation

To investigate whether reversible cell sonoporation was achieved at a single-cell level after TSAW device treatment, the calcein-AM (Sigma-Aldrich, St. Louis, MO, USA) and PI (Sigma-Aldrich, St. Louis, MO, USA) double-staining method was used to characterize the reversible perforated cells. Calcein-AM only stains living cells to generate green fluorescence. When the cell membrane is intact, PI cannot enter the cell freely. Once the pore is formed in the cell membrane, PI can enter the cell and bind to DNA and RNA, emitting red fluorescence. Therefore, the cell simultaneously emits green and red fluorescence, indicating that it has achieved a reversible perforation effect.

### 2.5. Bubble Cavitation Detection

LDV based on a PCD method was used to measure whether bubble cavitation exists during TSAW excitation [14,29]. A laser beam generated by LDV was focused on the oscillating bubble surface to detect the frequency spectrum signals. If harmonic signals or broadband noise emitted by the oscillating MBs could be detected, it proved that bubble cavitation has occurred.

### 2.6. Statistical Analysis

All data are expressed as the mean ± standard error of mean (SEM). An independent sample *t*-test and an analysis of difference were performed and employed for comparison between multiple groups. A value of *p* < 0.05 was set to be statistically significant.

## 3. Results and Discussion

### 3.1. Sonoporation at a Single-Cell Level

**Figure 2:**
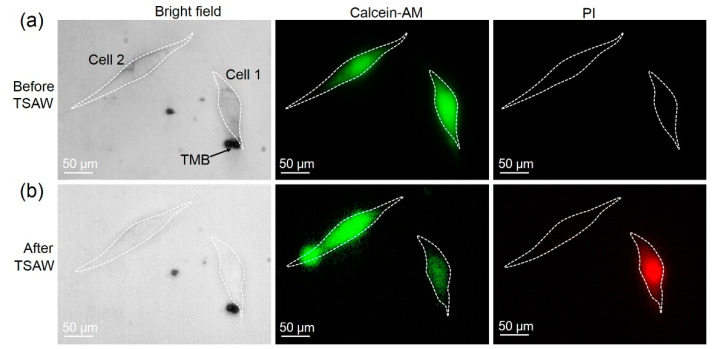
Sonoporation achieved at a single-cell level. (**a**) Adhesion of TMBs to cells and cell viability, before TSAW treatment. The bright field shows TMBs adhering to cell 1 but not to cell 2. Fluorescent images shows good cell viability of cell 1 and cell 2. (**b**) Sonoporation achievement of the cell adhering TMBs with TSAW treatment. The red fluorescence could be only observed in cell 1 and the green fluorescence still existed in cell 1 and cell 2.

The adhesion of TMBs to the cell surface is indispensable for the reversible perforation of cells. The input voltage of 350 mVpp was chosen to excite the TMBs, causing no obvious change in the radius of the TMBs. In Figure 2a, two TMBs adhered to cell 1, while no TMBs adhered to cell 2. Before TSAW stimulation, fluorescent images showed that cell 1 and cell 2 emit green fluorescence and no red fluorescence, indicating the good cell viability of cell 1 and cell 2. In Figure 2b, after TSAW treatment, cell 1 emitted both green and red fluorescence, but only the green fluorescence was observed in cell 2, demonstrating that small holes were generated in the cell membrane of cell 1 and PI could enter cell 1 and combine with the DNA and RNA to produce red fluorescence. Meanwhile, calcein-AM also flowed out of cell 1 through the small holes, weakening the fluorescence. Since the images were obtained 10 min after TSAW treatment, green fluorescence still existed in cell 1, showing that cell 1 was still alive.

**Figure 3:**
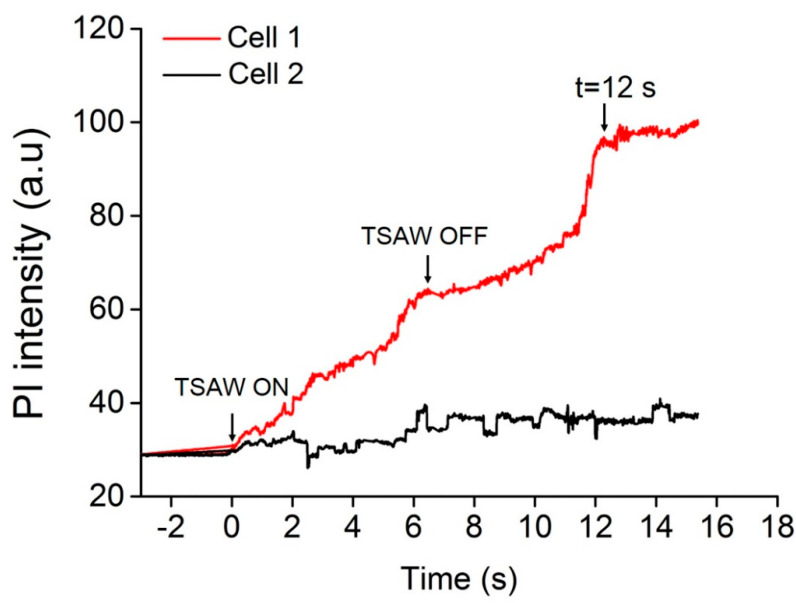
The curve of PI fluorescence intensity for cells with time. Before and after TSAW excitation, the increase in the rate of PI fluorescence intensity was different and the recovery time of a cell membrane pore might be 12 s.

Cell 2 was selected as a control. After ultrasound stimulation, cell 2 still had no red fluorescence, indicating the absence of small holes. The results verified that the TSAW device assisting the TMBs could achieve cell sonoporation at a single-cell level. Furthermore, during ultrasound excitation, the mean fluorescence intensity of PI in cell 1 and cell 2 was also analyzed. Figure 3 shows the mean PI fluorescence intensity in cell 1 over time using Image J software. It shows that the fluorescence intensity of cell 1 gradually increased with the TSAW stimulation time, while there was no change in the PI fluorescence intensity in cell 2. When the TSAW excitation was stopped, the PI fluorescence intensity still increased, but at a relatively lower rate. After 12 s, the PI fluorescence intensity did not increase, which proved that the small holes may have been repaired completely. This verified that the cell sonoporation induced by the TSAW device is a reversible process. 

**Figure 4:**
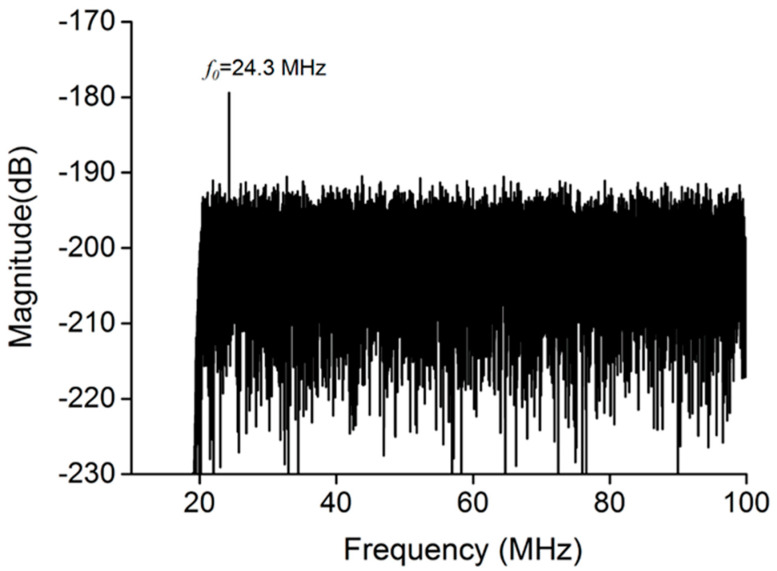
The measurement of bubble cavitation in the PDMS channel. No harmonic signal or broadband noise was observed, indicating that no bubble cavitation had occurred.

LDV based on a PCD method was employed to detect whether bubble cavitation occurred. Figure 4 shows that after TSAW excitation, no harmonic signals associated with stable cavitation or broadband noise along with inertial cavitation were observed, determining that no bubble cavitation had occurred. 

### 3.2. TMBs’ Translational Movement Induced by the ARF

**Figure 5:**
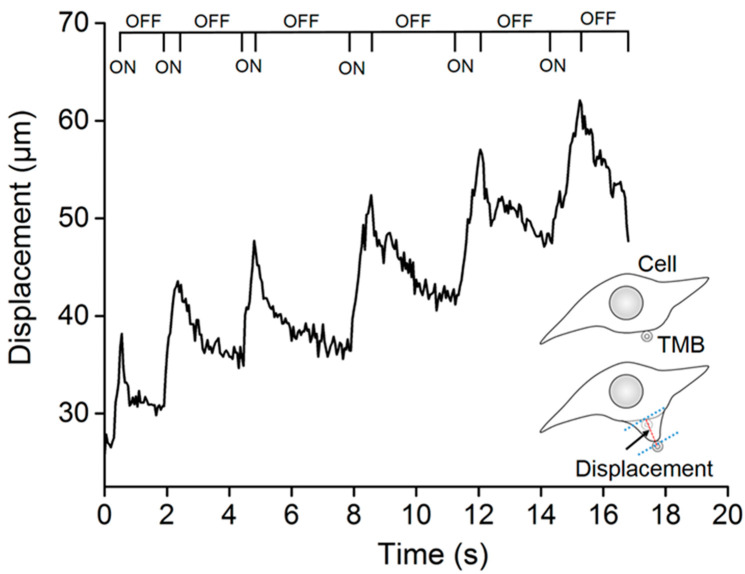
Effect of TSAW stimulation on TMB translation. After ultrasound excitation, the TMBs moved in the direction of TSAW propagation. Once the ultrasound stopped stimulating, the TMBs partially moved back to the original position.

To investigate the mechanism underlying cell sonoporation, a high-speed CCD (OptiMOS, QImaging, Canada) was employed to record the translational movements of the TMBs after TSAW stimulation. During the experiments, a sonication duration of 1 s, an inter-stimulus interval of 4 s, and a total time of 100 s were selected to excite TMBs. The TMBs were bound to MDA-MB-231 cells for the whole experiment. As shown in Figure 5, the TMBs adhering to the cell membrane were pushed away (pulse-on) in the TSAW propagation direction, and then pulled (pulse-off) toward their original position. Moreover, the translational distance of the TMBs became larger with the accumulation of the number of TSAW stimulations. When the TSAW stimulation was turned off, the TMBs could not completely move back to the last position and the time required was much longer. Several microfluidic studies have demonstrated that the translational movement of TMBs largely depends on the ARF in the ultrasound field. Furthermore, the TMBs being pulled (pulse-off) toward their original position might be attributed to the elastic properties of the cytoskeleton of the attached cells. 

### 3.3. Cell Sonoporation Induced by Cell Membrane Deformation

**Figure 6:**
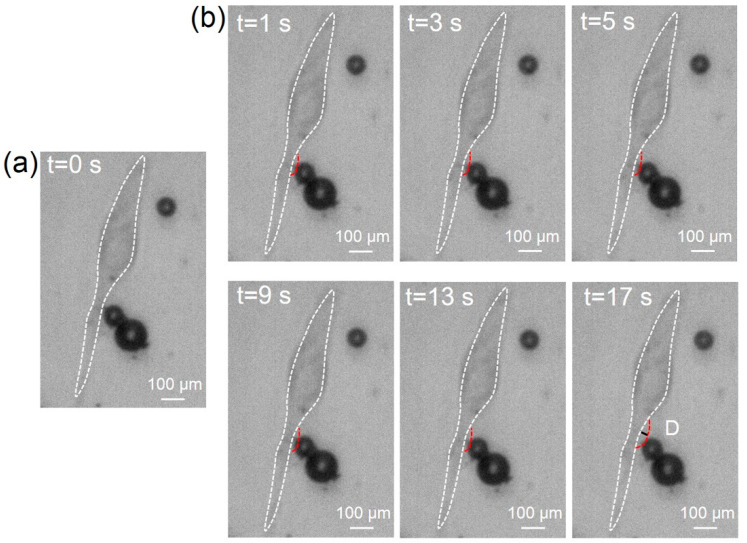
The deformation of the cell membrane during TSAW stimulation. (**a**) Optical image for original position of cells and TMBs. The TMBs tightly adhering to the cell during the whole process. (**b**) The deformation of cell membrane with TSAW excitation. The white dotted line represents the initial position of the cell, and the red dotted line shows the position of cell deformation in real time. D is the value of deformation at different times.

When the TMBs moved, a pulling and pushing force was generated at the cell membrane boundary where the TMBs were attached, due to the MDA-MB-231 cells adherently growing in the PDMS channel. Because the TMBs were bound to the MDA-MB-231 cells by biotin–avidin–biotin, the TMBs directly impelled the neighboring cell to perforate. In the whole experiment, the TMBs were tightly bonded to the cell. In Figure 6a, TMBs can be seen adhering to the cell surface before TSAW stimulation (t = 0 s). During the ultrasound stimulation period, the cell membrane in contact with TMBs was pulled in the same direction as the movement of the TMBs to the point of deformation, as shown in Figure 6b. When the TSAW excitation was stopped, the cell membrane tended to go back to its original position, moving in the direction of the ultrasound source. With the accumulation of TSAW stimulation, the deformation of the cell membrane gradually increased. Ultimately, cell membrane deformation occurring multiple times because of repeated pulling by TMBs led to cell sonoporation. Since Figure 4 verifies that no bubble cavitation occurred, it further proves that the mechanism of the TSAW device for cell sonoporation may mainly rely on the ARF acting on TMBs, propelling the cell to deform. 

### 3.4. Impact of Acoustic Input Voltage on Cell Sonoporation

**Figure 7:**
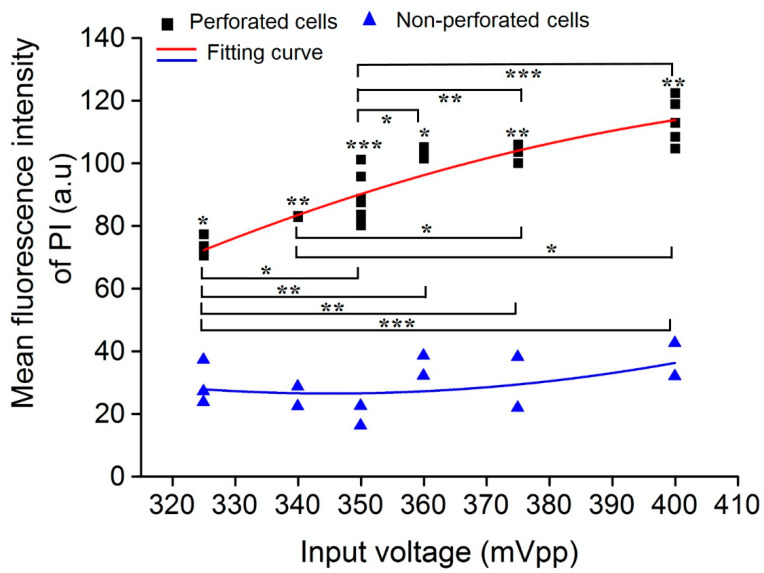
The impact of acoustic input voltage on cell sonoporation. The relationship between the ultrasonic input voltage and the mean PI fluorescence intensity. The degree of perforation was positively correlated with the input voltage. *** p < 0.001, ** p < 0.01, and * p < 0.05.

The acoustic input voltage had a key impact on cell perforation, and the relationship between the input voltage and perforation characteristics was analyzed. In the experiment, the influence of various ultrasound input voltages on cell perforation was evaluated when two TMBs adhered to the cell membrane. Figure 7 shows the PI fluorescence intensity distribution of perforated and non-perforated cells at various ultrasonic input voltages. It indicates that a significant difference of mean PI fluorescence intensity exists between the perforated and non-perforated cells at various input voltages. For perforated cells, PI fluorescence intensity showed a significantly statistical difference with the increase in the voltage, demonstrating that the degree of perforation was enhanced with an increase in the input voltage, which is significant for macromolecular delivery. When the input voltage was 350 mVpp, high cell sonoporation efficiency was achieved. Meanwhile, the linear fitting results show that the degree of perforation was positively correlated with the input voltage, while the non-perforated cells were not correlated. 

### 3.5. Impact of TMB Number on Cell Sonoporation

**Figure 8:**
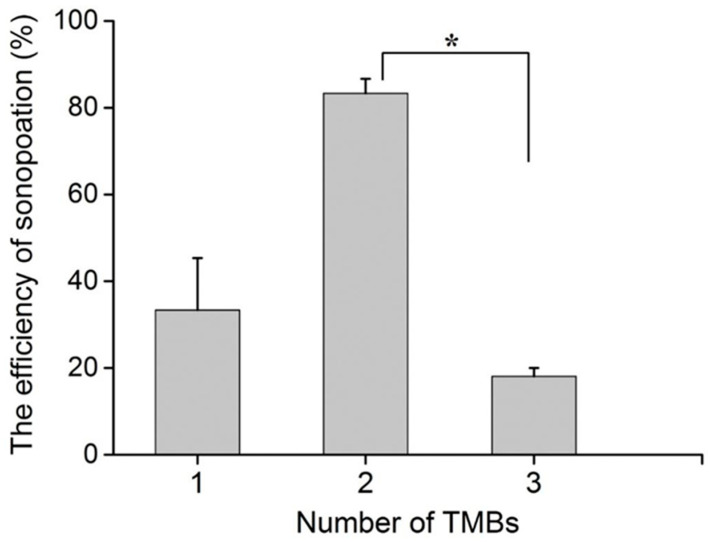
The impact of TMB number on cell sonoporation. When the ultrasonic input voltage was 350 mVpp, the cell sonoporation efficiency was significantly different for the two TMBs bounded to the cell membrane, compared to the other groups. * p < 0.05 and 10 cells were used for each group across the three experiments.

The number of TMBs was also an important factor affecting cell perforation. In the experiment, one, two, and three TMBs were selected, and the ultrasonic input voltage was set to 350 mVpp. Figure 8 shows that the highest sonoporation efficiency of 83% was achieved by two TMBs adhering to MDA-MB-231. In our experiments, aggregated TMBs were selected to evaluate the impact of TMB numbers on sonoporation efficiency. When three TMBs were attached to the MDA-MB-231 cells, they bonded together and acted as a larger bubble, compared to two TMBs. Therefore, the translational movement of TMBs may be shorter under the same input voltage, which eventually induced a smaller deformation of the cells. This may directly cause a lower sonoporation efficiency. The results demonstrate that the cell with two TMBs attached may be subjected to greater push or pull, causing a change in the permeability of the cell membrane and promoting the formation of small pores on the cell membrane. 

In fact, this TSAW device offers a suitable method to study the mechanism of cell sonoporation. On the other hand, its throughput is still limited by the non-precise control of the microbubbles. In our future work, a standing SAW (SSAW)-based acoustofluidic device will be proposed to precisely control the MBs and achieve a high throughput for cell sonoporation.

## 4. Conclusions

In this paper, a TSAW device based on non-cavitation TMBs was designed to achieve reversible cell sonoporation at a single-cell level. On ultrasound excitation, the TMBs attached to the cell membrane were subjected to an ARF, which pulled or pushed the cell membrane to the point of deformation and promoted the formation of pores on the cell membrane. The optimal parameters were an ultrasound input voltage of 350 mVpp and two TMBs adhering to the cell, bringing about a cell perforation efficiency of up to 83%. This TSAW device can serve as an efficient tool to study the mechanism of ultrasound bioeffects and can be potentially be applied in gene transfection.

## Data Availability

Not applicable.
